# Designing Culturally Adapted Digital Mental Health Support Tool for Chinese-Speaking International Students in Australia: A Qualitative Co-design Study

**DOI:** 10.2196/76695

**Published:** 2025-10-21

**Authors:** Ling Wu, Chen Zhu, Joshua Paolo Seguin, Jue Xie, Pranav Kulkarni, Mingye Li, Patrick Olivier

**Affiliations:** 1 Action Lab, Department of Human-Centred Computing, Faculty of IT, Monash University, 14 Rainforest Walk, Clayton, 3168, Australia, 61 03 - 9905 5859

**Keywords:** mental health digital tool, international students mental health, culturally-adapted resources, self-directed tool, co-design digital tool

## Abstract

**Background:**

International students, especially Chinese-speaking students, who grew up with collectivist values and the linguistic and cultural characteristics face a higher risk of mental health issues due to the challenges of geographical, linguistic, and cultural transitions when studying abroad. While digital technology has shown promise in supporting mental health, few studies have focused on designing tools specifically for Chinese-speaking international students and the challenges they face.

**Objective:**

This study aimed to design and develop a self-directed digital mental health support tool that provides culturally safe and appropriate support for international students.

**Methods:**

A co-design approach was used across 2 study phases, Phase 1 (interviews) and Phase 2 (co-design workshops), to explore the design implications of the digital tool. Inductive thematic analysis was conducted to extract design insights and considerations.

**Results:**

Findings show that participants faced a wide range of challenges arising from cross-cultural, academic, and daily life demands, which further contributed to increased levels of stress and negative feelings. These findings highlight a need to improve mental health awareness, literacy, and help-seeking intentions in this vulnerable group. These insights informed the design that emphasized the integration of culturally adapted resources with self-directed learning tools.

**Conclusions:**

Based on the findings, a personalized, self-directed, and culturally adapted design has been proposed that creates a bridging pathway linking students’ immediate challenges with mental health education and support. This design offers a clear set of implications for enhancing international students’ mental health awareness, literacy, and help-seeking behaviors, thereby providing essential support for this population.

## Introduction

### Background

Over the past 2 decades, international student mobility has surged, with over 6.8 million international students worldwide as of 2022 [1] and Australia emerging as a popular destination, with more than 810,960 international students recorded by May 2024 [2]. Chinese-speaking students account for roughly 20%-30% of these enrollments, and many face significant challenges in adapting to the new cultural and academic environments, accompanied by varying levels of mental health issues. Specifically, Chinese-speaking international students have reported a higher risk of experiencing mental health issues, such as depression, anxiety, and stress compared to Australian domestic students [3,4].

A wide range of factors, including language barriers, communication challenges, and social difficulties—often influenced by cultural differences, particularly the collectivist values of Chinese-speaking international students and the language barriers—significantly contribute to the development of mental health issues [4-6]. These factors, coexisting with daily living challenges [7,8], low levels of mental health awareness and literacy [9], and exposure to mental health stigmatization rooted in collectivist values and filial piety in China [10-12], further exacerbate the limited attempts of help-seeking and the underuse of mental health services in Australia.

While universities are typically the first point of contact for international students facing mental health issues, a study revealed that their mental health policies, strategies, and educational initiatives for international students are either lacking or framed as largely a responsibility of the student [13]. This creates significant challenges, especially for high school and undergraduate students who are at a developmental stage (ie, late adolescence, typically of age 15‐19 years), making them more vulnerable to mental health issues [14].

### Existing Digital Tools

Several digital mental health resources in Australia offer valuable support for university students [15-18], particularly those international students from Chinese-speaking backgrounds. The MindYourHead platform (University of Sydney) delivers bilingual self-assessments on stress, sleep, procrastination, and social support, using culturally resonant terms, such as “beng kui” (ie, mental breakdown) to enhance user engagement [19]. While this linguistic localization strengthens accessibility, the tool has tended to focus on emotional and sleep-related concerns and does not address broader, multifaceted stressors, such as academic workload, visa uncertainty, financial pressures, and social transitions. These are the major areas often noted by students as affecting their well-being. High dropout rates also suggest that further engagement with the users may be needed to maintain sustained use.

Meanwhile, This Way Up, a collaboration between St Vincent’s Hospital Sydney and UNSW, delivers structured, self-paced internet-based cognitive behavioral therapy courses for conditions like anxiety and depression [20]. The platform has been rigorously evaluated and shows strong outcomes across general populations; however, some challenges remain. As with many self-guided programs, attrition rates can be high, and opportunities for personalization are limited, with content delivered in a fixed, workbook-style format. Although multilingual resources, including simplified and traditional Chinese, have recently been introduced, these adaptations are broad and not specifically designed to address the cultural, academic, and social challenges experienced by Chinese-speaking international students.

Moreover, the Chinese Online Clinic, developed by the Black Dog Institute, offers a free and anonymous mental health self-assessment fully translated into Mandarin, providing evidence-based personalized feedback and resources [21]. This tool reduces language barriers and increases accessibility for Chinese-speaking users, though it primarily targets the general population and does not explicitly address the specific academic, multicultural, and transition-related contexts of university students. In a broader context, systematic reviews of digital mental health interventions for university populations have highlighted persistent gaps. While these tools show potential to improve access and deliver evidence-based support, many are characterized by high dropout rates, limited personalization, low engagement, and user concerns about credibility and interface quality [15-18]. Together, these findings suggest that despite meaningful progress, current digital interventions often fall short of addressing the specific cultural and developmental needs of Chinese-speaking international students.

### Study Aims

To address these limitations, this study engaged Chinese-speaking international students in Australia in the co-design of a digital mental health support tool. The overarching aim was to create a free, culturally safe, and self-directed platform that connects students’ everyday challenges with mental health literacy and support options. Phase 1 focused on developing the tool’s content through qualitative analysis of students’ lived experiences, while Phase 2 refined both the content and the interface based on user testing of an initial prototype. By grounding the design in students’ own perspectives and iteratively improving usability, this study represents the first steps toward a tailored intervention that can enhance awareness, promote safer coping strategies, and facilitate pathways to professional help when needed.

## Methods

### Recruitment

The current paper covers Phase 1 and Phase 2 of a broader study on the design, development, implementation, and evaluation of a digital mental health support tool for Chinese-speaking international students. The study follows the Double Diamond design framework [22], with Phase 1 and Phase 2 representing the Discover and Define stages, respectively. Specifically, Phase 1 aims to discover existing mental health needs (eg, academic, everyday life, and social activities–related challenges experienced by Chinese-speaking international students in Australia) through semistructured interviews and use the insights extracted to design a prototype. Phase 2 aims to further define the key aspects of the intervention (content focused) and key functionalities (feature focused) of the platform relevant to international students that shaped the final design of the prototype.

Participants in Phase 1 were recruited between August and October 2023 through internet-based advertisements targeting Chinese-speaking international students who were interested in addressing their mental health concerns while studying in Australia and contributing to the development of a digital tool. The target population was defined as current international students holding a valid student visa, which inherently excluded Australian citizens, permanent residents, and holders of other visa types (eg, working visas). Potential participants were screened using the information provided in the expression of interest sign-up form according to the following inclusion criteria: (1) current international students enrolled in a higher education degree in Australia, (2) studying onshore, and (4) native speakers of Chinese. Individuals were excluded if they were less than 18 years of age or were experiencing a mental health crisis (ie, self-reported acute risk or severe psychiatric symptoms requiring urgent assessment or care that could compromise informed consent or safe participation) at screening. Given that the intended users of the platform are international students who may present with varying levels of mental health needs and mental health literacy, no additional exclusion criteria beyond those specified were applied. The Depression, Anxiety and Stress Scale–21 items (DASS-21), embedded in the prototype, provided a standardized means of assessing psychological distress, offering additional context for interpreting participant responses. Information on participants’ length of stay in Australia was also collected to help situate their experiences within different stages of adaptation to the host country.

Eligible participants were contacted via email after filling out the expression of interest form voluntarily. In total, 20 participants took part in individual semistructured interviews during Phase 1. Phase 2 involved a total of 7 participants, allocated into 2 separate design workshops based on individual preferences and availability. Participants were drawn from Phase 1 individuals who expressed interest in further involvement and from internet-based advertisements targeting Chinese-speaking international students interested in testing the prototype. The same inclusion and exclusion criteria were applied throughout.

### Materials

During the semistructured individual interviews of Phase 1, a flexible set of open-ended guiding questions was used to explore the mental health needs and concerns of Chinese-speaking international students. While the core questions addressed areas, such as daily life challenges related to academics, living conditions, physical health, and emotional well-being, the sequence and specific follow-up questions varied depending on participants’ responses. This approach allowed the interviewer to probe deeper into emerging topics and clarify issues as they arose. Additional discussion points included how participants addressed these challenges, their help-seeking attitudes, and self-management capabilities. Participants were also invited to share their understanding of mental health issues, strategies for self-management, and preferred avenues for professional assistance in order to identify potential stigma and levels of mental health literacy. Finally, participants were asked about the digital platforms they commonly used for mental health information to inform the potential needs and features of a future digital solution. The interview guide is provided in the Multimedia Appendix 1.

In Phase 2, two separate design workshops were conducted in a group format, with 3 participants in workshop 1 and 4 in workshop 2. Before the workshop, participants were invited to interact with the prototype for 1 week at their own pace. They were encouraged to take notes. During the workshop, group activities were facilitated using a Miro board (RealtimeBoard, Inc [23]) to elicit participants’ feedback on the user journey of the digital support tool prototype. The design activities included a journey map [24], with a goal to understand the barriers and enablers at each touchpoint of the user journey. The activities focused on three key aspects: (1) the design of the mental health check-in, including surveys and questionnaires; (2) the system’s recommended content based on the survey and questionnaire results; and (3) the interface and functionalities. All participants opened the Miro board page on their own devices and acted as users to complete the collaborative activities. Engagement features, such as emoji reactions, thumbs-up indicators, and polls, were used to maintain engagement throughout the videoconferencing (Zoom). The workshop guide is provided in the Multimedia Appendix 2, where an example screenshot of the Miro board activity is also included.

**Figure 1.**
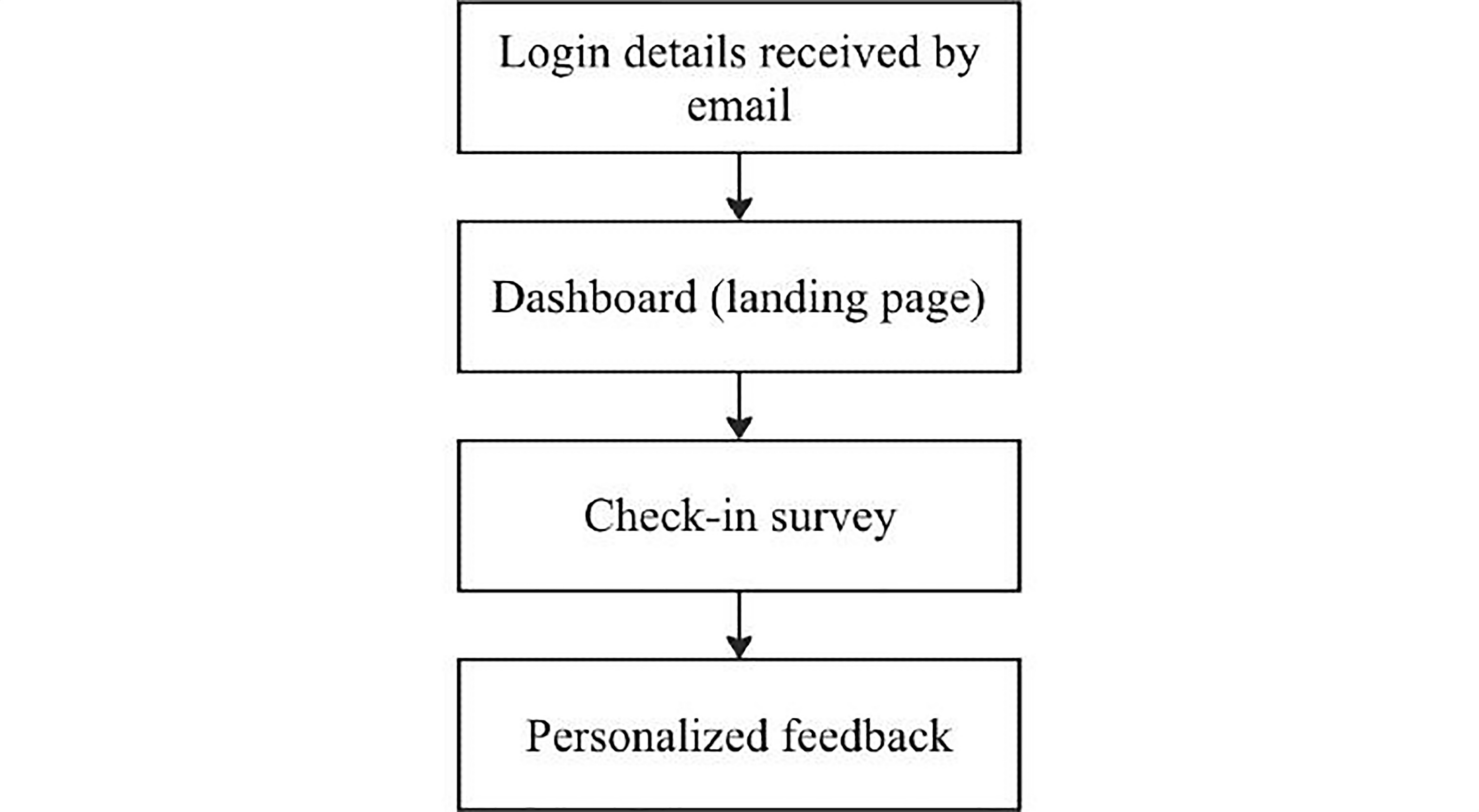
User flow diagram for the digital mental health support tool prototype.

The user flow of the digital support tool prototype is shown in Figure 1. Specifically, a check-in survey was embedded to explore the challenges faced by participants, followed by the short-form version of the DASS-21 [25] to assess their level of depression, anxiety and stress. As guided by the literature and findings from Phase 1, mental health issues among international students have been linked to a broad range of challenges, including those related to study, accommodation, language, social connections, finances, and lifestyle-related health factors. These domains informed the design of the check-in survey and were used to generate individualized feedback, ensuring that each user received tailored support addressing their specific needs. The individualized feedback also incorporated participants’ scores on each DASS-21 subscale, along with evidence-based strategies and resources, such as internet-based and local services providing mental health support in Chinese. Participants were able to download their complete feedback and leave any additional comments on the feedback page. They could also log back into the platform at any time to review their feedback.

The prototype was developed using TypeScript (Microsoft Corporation) and Gatsby.js (Gatsby, Inc) to deliver a fast and responsive user interface. Firebase (Google LLC) was used for authentication, database management, and hosting, while SendGrid (Twilio Inc.) was used for automated email communication. Zapier (Zapier Inc) facilitated workflow automation between services. The administrative interface was implemented with Next.js (Zapier Inc) to enable efficient management of content and user data. The interface design encompassed layout, color scheme, and other visual elements, with representative screenshots provided in the Multimedia Appendix 3.

DASS-21 [25] is a self-report scale designed to measure the negative emotional states of depression, anxiety, and stress through 3 subscales. Each statement is rated on a 4-point Likert scale ranging from 0 (“Did not apply to me at all”) to 3 (“Applied to me very much, or most of the time”), indicating how much the statement applied to the respondent over the past week. The score for each subscale is calculated by summing the item scores and then multiplying the total by 2 to compare with established severity cutoff scores. A higher score indicates a higher level of the corresponding emotional state. Both the English and Chinese versions of the DASS-21 have demonstrated good psychometric properties in university students [26,27].

### Procedure

During registration for both Phase 1 and Phase 2, participants electronically signed a consent form followed by a brief demographic survey (covering age, educational background, gender, and duration in Australia), via REDCap (Research Electronic Data Capture; Vanderbilt University) [28,29] hosted at Monash University. REDCap is a secure, web-based, and confidential platform for the collection of research data, allowing flexible data manipulations and export procedures. Eligible participants were subsequently contacted via email to determine their preference for attending interviews or workshops. All of them opted for the videoconferencing (Zoom) format. The interviews lasted approximately 60 minutes, while the workshops ran for around 90 minutes. All sessions were conducted via video conference and recorded, with transcripts prepared and translated into English by trusted third-party services prior to analysis.

### Analysis

The data analysis for the semistructured interviews and design workshops was conducted in NVivo (Lumivero [30]) using inductive thematic analysis. Two of the authors (LW and CZ) performed inductive thematic analysis independently, following the 6-step analysis pipeline described by Braun and Clarke [31]. An inductive process was adopted in the development of themes. Descriptive statements were made for each code, and themes were derived from the descriptive statements. This process was cross-checked and discussed by other members of the research team before the results were finalized.

### Ethical Considerations

This study (Phases 1 and 2) was approved by the Monash University Human Research Ethics Committee (Approved project ID 39583). The approved protocol covered recruitment materials, consent procedures, the conduct of videoconference-based interviews and design workshops, and the use of third-party services for transcription and translation, as specified in the ethics application.

Informed consent was obtained electronically via REDCap (Vanderbilt University, Nashville, TN, United States) before participation. Participants were informed about the study purpose, procedures, data handling, and their right to withdraw at any time without penalty.

All data collected were downloaded and stored with password protection on Monash University–managed secure servers, and access was restricted to investigators named in the ethics protocol. Data backups were maintained on network drives managed by Monash eSolutions. Identifiable information was removed from transcripts prior to analysis to ensure privacy and confidentiality.

Compensation was provided in the form of an AUD $20 (US $12.98) electronic gift card per hour of participation, as outlined in the approved ethics protocol.

## Results

### Phase 1: Interviews

#### Overview

For Phase 1, a total of 23 participants registered their interests, and 20 were included in the interviews and analysis. Three participants withdrew from the interview process. Demographic information was displayed in Table 1. From inductive thematic analysis, 3 themes and 27 subthemes were derived from 152 codes. Detailed descriptions and reference frequency of themes and codes are shown in Table 2.

**Table 1.** Demographic information of participants involved in Phase 1 (N=20).

Demographic variable		Participants
Age (years), mean (SD)		19; 23.79 (2.44)
Sex, n		
Female		13
Male		7
Nonbinary		0
Education, n		
Bachelor’s degree or diploma		9
Master’s degree		7
Doctoral degree		4
Study areas, n		
Commerce		4
Public health		8
Computer science		4
Social science		3
Years in Australia, n		
<1		3
1-3		6
3-6		8
>6		2

**Table 2.** Summary table of themes and subthemes derived from semistructured interviews.

Themes and subthemes		Code frequency (%)	
Student life challenges		207 (27.02)	
Coursework challenges		65 (8.22)	
Perceived heavy coursework load and difficult learning content or class activity lead to negative mental health states.		40 (5.22)	
Comparison with peers and uncertainty for the future lead to stress.		16 (2.09)	
Academic transition challenges (eg, different study mode or paradigm)		7 (0.91)	
Cross-cultural challenges		59 (7.70)	
Different values, beliefs, and habits lead to perceived discrimination, misunderstanding, and negative impact on mental health.		16 (2.09)	
Language barriers lead to poor academic performance, in-class interactions, and negative mental states.		43 (5.61)	
Daily life challenges		85 (11.10)	
Developmental stage–related challenges (eg, struggles related to self-efficacy and autonomy, self-regulation or management skills, and facing judgment).		23 (3.00)	
Logistical challenges (eg, transportation and accommodation) lead to negative mental states.		29 (3.79)	
Socializing difficulties lead to boredom and loneliness, thus affecting mental health.		33 (4.31)	
Lived experience of mental health issues		221 (28.85)	
Contextually shaped attitudes toward mental health		114 (14.88)	
Attitude toward mental health is more open and positive in the host country (ie, Australia), which encourages help-seeking.		35 (4.57)	
Attitude toward mental health is less open and negative in the home country (ie, China), which discourages help-seeking.		70 (9.14)	
Personal view of mental health is mixed but has changed to be more positive since arriving in Australia.		9 (1.17)	
Common mental health issues		66 (8.62)	
EMO^a^, anxiety, depression, and stress are common.		52 (6.79)	
Mental health issues can have significant influences on daily life.		14 (1.83)	
Mental health resources		41 (5.35)	
Interested in exploring self-management and help-seeking options		17 (2.22)	
Limited resources to learn about mental health		24 (3.13)	
Coping and managing strategies		338 (44.13)	
Concerns and perceptions about clinical services		127 (16.58)	
Limited help-seeking intentions for professional help		88 (11.49)	
Limited knowledge related to local health care systems		19 (2.48)	
Worries and negative perceptions about clinicians or services can discourage help-seeking.		20 (2.61)	
Common practice and different views on seeking support from peers (preferably) and parents (limited attempts and varies across individuals)		63 (8.22)	
Perception and experiences of online resources		52 (6.79)	
Negative perception of (eg, not trusting) online resources highlights the need for reliable resources.		29 (3.79)	
Positive perceptions of online resources (eg, immediacy, convenience, and efficiency)		23 (3.00)	
Some lifestyle strategies or activities reflect maladaptive coping.		47 (6.14)	
Some self-management strategies or tools are inappropriate, reflecting poor mental health literacy.		49 (6.40)	

a EMO refers to the equivalent of mood swings in English, encompassing sudden feelings of sadness (often occurring at night) or emotional responses during specific days or times, sometimes accompanied by anxious and depressive thoughts and feelings.

#### Student Life Challenges

The findings reveal that student life challenges arising from geographical and academic transitions are highly impactful for Chinese-speaking international students, who reported heightened stress levels and a noticeable decline in mental health. In addition to the acute multilevel transition (ie, location, culture, academic expectations and workloads, and social connections), these students also enter a level of independence that they might not have been sufficiently prepared for. As articulated in previous literature, high-level parental involvement in children’s and emerging adults’ lives is a common cultural phenomenon among Chinese parents [32] as a result of heavy influence from Confucian values and a collectivist cultural orientation [33]. With key emphasis on parental control and training (guan)—obedience, discipline, and respect; focus on academic achievement, a highly protective approach, and high parental involvement (Sun and Mulvaney [32]; Wu et al [33]). Therefore, transitioning from a familiar, high-support environment to an uncertain environment with close to no parental support can be highly challenging.

The most impactful and prevalent challenge faced by Chinese-speaking international students is cultural transition, including acute cultural adaptation issues, language barriers, and identity conflicts. These challenges can happen in various forms of lived experiences, such as perceived discrimination resulting from different cultural expectations, communication difficulties, and perceived social exclusion and isolation. These experiences tend to lead to feelings of isolation and a sense of *“*I don’t belong here” (Participant 8; P8*)*. In addition, students tend to perceive that they have inadequate proficiency in English that affects their academic performance and interpersonal interactions in social and studying settings. The consequence of this perceived inadequacy, together with the cultural differences, can lead to self-dissatisfaction, stress, and frustration, with no clear solutions to address these issues.

*As a non-native English speaking international student, you feel a bit discomfort, a bit difficult to fit in...for example, when they tell jokes, you completely don’t get their laughing points, and you just can’t really fit in their culture...Then, you feel a sense of being marginalized*.

[P12]

*I feel very frustrated because my spoken language is really poor... I’m just very dissatisfied with myself, and in such cases, there is a lot of psychological pressure*.

[P6]

Participants identified heavy study workload as a major challenge faced by international students. The language and cultural barriers often exacerbate these issues, making it significantly difficult to manage and cope with workload, with 1 participant describing the situation as:

*I might have to put in many times more effort than they [native English-speaking students] do to cover the gap [caused by language barriers*].

[P12]

They articulated many other difficulties in managing and coping with the demands of participating in group discussions, adapting to new learning environments, passing subjects, completing assignments, and understanding class content. They have also identified some perceived psychological impacts. For example, they can feel behind with their academic performance, resulting from peer pressure and constant comparison with their peers (P2), which further increased their anxiety about future educational and career plans. Other participants shared that a sense of uncertainty often led to negative feelings, such as confusion, stress, self-doubt, and anxiety.

*After starting university, I feel extremely stressed. The teaching model here, although the tutorials are only two hours, it actually requires more than five hours of preparation and review outside the class time...I often feel on the verge of breaking down and regret choosing this major*.

[P21]

*There would be feelings of inferiority. It might include comparisons with peers. It could be comparisons of abilities, academic performances, and maybe even appearances, possibly. Family backgrounds, financial conditions, all sorts of comparisons stemming from peer pressure*.

[P2]

The most commonly shared daily life challenge is difficulty with socializing. Common problems include worries about, or challenges in, establishing intimate relationships, making new friends, or formulating a social network. Alongside academic challenges, these social and interpersonal issues are seen as significant sources of stress, which can further exacerbate feelings of loneliness and isolation, significantly impacting their well-being. Challenges related to logistical life management are another primary cause of negative feelings, as many of the participants did not have adequate prior experience living independently in their home country to prepare themselves. These challenges vary and may include financial burdens, lengthy transportation, homesickness, accommodation issues, and unfamiliarity with social rules or systems. Specific problems, such as living in a remote area due to cost of living concerns (P8), sleep deprivation due to accommodation issues (P21), inefficient transportation systems (P19), and not being able to understand and meet cultural expectations (P16), can lead to feelings of stress, anxiety, and depression. These difficulties can be particularly challenging for new international students due to the drastically different living arrangements between their home country and the host country, especially with no immediate support or solutions available to address them.

*I often feel very lonely, especially since I feel like many students here might not be very eager to make friends, I think it is a kind of pressure to make new friends*.

[P8]

*The noise problem where I lived had a significant impact on my life. I didn’t know how to communicate about it, and I really wished someone had guided me on how to handle the situation. My parents [who were in China] didn’t know what to do, and neither did I, as I was only 15 years old at the time*.

[P21]

#### Perceptions and Lived Experience of Mental Health Issues

Participants expressed varied views and attitudes toward mental health issues from their home and host cultures. They described that they see a more open attitude toward mental health and that mental health is more normalized in their host country. Their perceived attitudes toward mental health were that it is “common in daily life” (P1), and people are “open to talking with others” (P16) and “having access to more professional mental health services” (P18). In contrast, participants’ perceptions of attitudes toward mental health in their home country were that there was stigma around it. These views are consistent with the literature demonstrating that collectivist cultural values (including Confucian influence) inform stigmatizing beliefs about mental illness [34,35]. Key stigma types are public stigma, where fear of damaging family reputation and disrupting social harmony leads to the public viewing mental illness as a moral weakness [35], and self-stigma, where individuals internalize culturally informed stigma beliefs, resulting in low self-esteem and shame [34]. Consistent with these studies, participants in our study perceived that this attitude led to “mental health issues are not being taken seriously” (P9), people “feeling shame over having mental health issues” (P14), and people being “unlikely to seek or talk about support with others” (P18) and more likely to be “at risk of facing discrimination” (P16). In general, the perception is that mental health, especially issues and disorders, is something that people tend to keep to themselves, sometimes not even shared within their family.

*Under the influence of their parents, they might feel that it is shameful or difficult to speak about...but I’ve found that many Chinese people are willing to share about their mental health issues once they are abroad. However, if they are in China, speaking out might lead to discrimination or being viewed differently. Therefore, they might be afraid to express themselves, and they also might not dare to talk to their parents about it, or if they do, they might receive a negative response*.

[P12]

When asked about their own experience of mental health, most participants stated that they think certain signs and symptoms related to mental health are prevalent among Chinese-speaking international students, including themselves. The most frequently reported issue (16 out of 20 participants) the participants articulated was “emo” the equivalent of mood swings in English, including sudden feelings of sadness (often happening at night) or emotional responses during specific days or times, some followed by anxious and depressive thoughts and feelings. These experiences were often attributed to homesickness (P16) and stress related to assignments and exams (P22), as well as social isolation (P18) and sometimes uncertainty associated with their future plans. For some participants, these temporary negative moods impacted their daily functioning, resulting in symptoms, such as insomnia and reduced appetite (P14) or a desire to be alone (P4).

*I think it might last for a long time. The feeling of being* “*emo” is like having small bouts of negativity, where life seems to lack hope… Life feels pretty dull, with no motivation… and this feeling can actually last for quite a while. Sometimes, it might even last the whole winter*.

[P23]

When sharing their perceptions and experiences of mental health, participants voluntarily mentioned a variety of sources to help understand their mental health–related experiences, including books, friends, university coursework, and informational sheets. A few participants also indicated a need to acquire this knowledge but were not sure about the ideal sources. Among the types of knowledge they were interested in, self-management strategies were most commonly mentioned (10 out of 20). Participants were keen to learn how to manage negative moods, create study plans, and obtain personalized support. A small number of participants expressed interest in finding systematic guidelines because they had not interacted with a professional before.

*Topics related to psychology, like mental health and how to manage emotions, are the ones I am interested in. I’ve always been very interested in how a person can maintain a positive attitude continuously*.

[P23]

*Because I haven’t seen a psychologist myself, most of my knowledge comes from what I’ve heard from others. I might not be clear on some of the details. If there were a systematic guideline, I believe it would be more helpful*.

[P1]

#### Coping and Managing Strategies

The overall approach to coping and managing their mental health articulated by the participants is self-directed, with some willing to use their family and social networks and a very small number of participants open to professional help, whereas the majority are resistant. This approach is again consistent and grounded in the Chinese cultural beliefs. With the feeling of shame and guilt resulting from not meeting culturally accepted expectations and behaviors, individuals tend to source self-directed and confidential means of mental health support [36]. With a family-oriented health system [37], help from the family and close relatives and friends may be possible for some, while others may experience heightened stigma and isolation due to not wanting to burden or bring stigma toward the family.

Participants discussed several strategies they use to manage their challenging feelings and moods, and the use of online resources is reported by all participants. They frequently turn to social media platforms and websites for assistance. For the students, these online platforms provide immediate and helpful information in allowing them to learn from others’ experiences, identify people with similar issues, better understand their own symptoms, and explore topics of interest. While participants found social media platforms, alongside other information sources, helpful to some degrees, they also articulated a concern that it is hard to judge if the information is reliable and trustworthy. Consequently, some participants expressed that if possible, they would use university-authorized online platforms, as they perceive a higher likelihood of trustworthy information there.

*Because you don’t understand the symptoms, you definitely need to observe yourself first. We don’t know what these symptoms could indicate—what disease or behavior. But you can start by searching online to see if there are similar symptoms. That’s how you might begin to understand what the issue could be. Then, you can further your understanding by listening to discussions on platforms like RedNote [a Chinese-based social media platform that enables users to share personal posts, like Instagram], reading comments, or learning about similar experiences and how others have resolved their issues*.

[P7]

*Because it lacks a certain level of professionalism, most of it is just individuals sharing their personal experiences, so it might not be very prominent in terms of professionalism. It’s really just sharing personal experiences. Yes, I understand that as an individual, I’m still looking for a more authoritative academic resource to learn about psychological knowledge*.

[P1]

Other than online resources, participants also explored various offline management options. The majority of the strategies used are mostly of a self-directed or self-organized nature. The most frequently used strategies included self-regulated strategies, engaging in conversations with friends and family, adopting lifestyle strategies, and joining entertaining or outdoor activities. Self-regulation strategies included (1) “just accept it” (P8); (2) “befriending negative moods” (P5); (3) better time management or listening to lecture recordings when negative moods were linked to academic pressures; (4) improving language skills to overcome language-related stress; and (5) using mental health techniques, such as shifting attention, practicing mindfulness, or meditation. A few participants mentioned that being alone and processing emotions internally is their preferred way to cope. In relation to lifestyle adjustment, participants listed various lifestyle changes and activities they use to manage their negative moods. These strategies cover “getting more sleep” (P4), “engaging in physical activities” (P3), “writing in a diary” (P16), “watching videos” (P17), “hanging out with friends” (P7), and “eating favorite foods” (P11). However, some participants also mentioned coping strategies, including playing video games and drinking alcohol.

*Like drinking and playing video games, I think these are ways of escaping from reality*.

[P8]

Interestingly, participants’ views on seeking social support within their circle are divided. Some participants felt that discussing their feelings with parents would not be appropriate or helpful, especially since many parents were located in China and may not fully understand their experiences. Moreover, a desire to be independent, to avoid worrying others, and not wanting to burden others were cited as reasons why some participants preferred not to discuss their issues. In contrast, many participants articulated that talking with friends (P12; P23; P5) or parents (P14; P22; P3) could be beneficial, as it provides opportunities to express and release negative emotions, feel understood, receive support, and relax, particularly when peers are experiencing similar issues related to academic stress. These findings suggest that, if available, students tend to primarily seek help from within their social circles. When this social circle is not available or not trusted by the participants, they tend to turn to a self-support approach where they manage things alone.

*I feel like they (parents) don’t understand a lot of things. Especially about life abroad—if you tell them what happens overseas, it’s completely unhelpful as they are utterly unaware and know nothing about the various situations and life conditions abroad. Telling them, I feel, has no effect at all*.

[P23]

*I won’t tell others everything, but the most likely time I might share these feelings is at night. At night, when I really feel overwhelmed and can’t bear it, I might think about talking to others. However, I don’t know why, perhaps it’s my independent and strong-willed nature that prevents me from actually doing so, and I end up preferring to endure it on my own*.

[P18]

While a few participants expressed interest in seeking professional support, most shared resistance and concerns toward taking action on seeking professional help. The most common concerns include language and cultural barriers and worries about costs, followed by appointment availability, the cultural and professional background of the clinicians, and the location of the services. Moreover, many participants indicated that they would seek help from clinical services only if they experienced obvious mental health issues. They (11 out of 20) also noted that they have very limited knowledge about the Australian health care system, international student health insurance policies, and accessing general practitioners. There was also the perception that help-seeking is more applicable when someone has severe mental health problems, and a few of them expressed concerns about accessing these services, indicating distrust and resistance toward clinicians and health care services.

*Some of the available counselors speak only English, and I would prefer to speak with a Chinese person because there are many specific terms that are unique to our culture. Right now, I can’t think of any specific examples, but terms like Neihao [internal friction, self-sabotage, or mental and emotional exhaustion caused by internal conflicts or overthinking], wuligan [a feeling of powerlessness or helplessness], I wouldn’t even know how to explain these to them. That’s why I would prefer to find a counselor with a similar cultural background*.

[P15]

*So I feel that my symptoms are not severe enough to seek this treatment. I think it’s only when the illness is at its worst that I would consider it*.

[P7]

*But my concern is that, after I speak up, I worry about the psychologist’s opinion of me because the things that trouble me are quite private. I’m also worried about the judgments from others. Even though I know that psychologists are professionally trained not to judge their clients, I still feel a bit anxious about this*.

[P21]

#### Phase 1 Design Insights and Strategies

The Phase 1 interviews demonstrated that Chinese-speaking international students face substantial academic, cultural, and daily life challenges that often give rise to stress, anxiety, self-dissatisfaction, and withdrawal. These difficulties, however, were seldom described by students in terms of “mental health,” but rather as the expected burdens of studying abroad. This disassociation suggests that many students do not recognize the connection between their everyday struggles and broader mental health concerns. Interventions therefore need to be anchored in students’ immediate experiences, framing common difficulties with coursework, language barriers, and social integration as meaningful signals of well-being. Embedding mental health concepts into resources that address these concrete stressors may enable students to identify early warning signs of poor mental health [38] in a manner that reduces stigma and increases relevance.

While participants perceived that mental health is discussed more openly in Australia than in their home country, they also acknowledged that stigma rooted in Chinese cultural norms continues to influence their own responses. This internalized stigma contributed to a tendency toward self-reliance, with students preferring to manage problems privately rather than seek professional assistance. Familiar expressions such as “emo” were used to describe low mood or stress; however, students rarely linked these experiences to mental health frameworks. Culturally sensitive interventions that recontextualize these expressions within an evidence-based understanding of mental health can begin to shift perceptions. Such an approach is consistent with Jorm’s framework on mental health literacy, which emphasizes improving knowledge and beliefs about mental disorders to facilitate recognition, management, and prevention [39,40].

The coping strategies reported by participants were predominantly self-directed, ranging from lifestyle adjustments and online searching to informal peer support. While some of these strategies were constructive, others, such as excessive gaming or alcohol use, carried risks. Concerns about the credibility of online resources were common; however, students continued to rely on them due to immediacy and accessibility. Professional services were generally viewed as relevant only for severe cases and were hindered by language barriers, cost, and mistrust. These findings indicate a need for interventions that provide trusted, university-endorsed resources, promote safer self-management, and create low-threshold entry points to care. Incorporating culturally aligned recommendations, university-endorsed supports, or peer-led information may help build trust and encourage gradual engagement with professional services [41].

In summary, Phase 1 findings highlight the importance of culturally sensitive, staged interventions that validate everyday struggles, foster mental health literacy, and strengthen pathways to appropriate support, in line with Jorm’s mental health literacy framework [39,40].

### Phase 2: Co-Design Workshops

#### Overview

For Phase 2, a total of 7 participants who had interacted with the initial prototype for a week were included in the design workshops, with 3 and 4 participants allocated to 2 separate design workshops, respectively. Among them, 4 had participated in Phase 1, and 3 were recruited through internet-based advertisement. The thematic analysis of the journey mapping and “I like, I wish, what if” design activities elicited themes related to positive (“Current strengths”) and constructive (“Areas to improve”) feedback and alternative ideas on the overall content. Textbox 1 shows the participants’ feedback on the 3 main topics of discussion, namely specific survey question development, recommendation and feedback content (based on survey results), and functionality and interface design.

Textbox 1. **Design considerations derived from group design workshops in Phase 2.**

**Survey and questionnaire content development**

- Areas to improve
  - Wordings or specific terminologies (eg, self-efficacy) were not easily understood
  - Questions from other scales and incorporating different dimensions of mental health issues
  - Questions need to be more comprehensive and could cover career and study planning, collaboration skills, financial management, family commitment, and visa issues
- Current strengths
  - Depression, Anxiety and Stress Scale–21 items (DASS-21) is a reliable tool
  - Questions cover a range of different daily life circumstances

**Func** **tionality and interface des** **ign**

- Areas to improve
  - Language option for the resources from external links
  - Online forum to share experiences or thoughts
  - Option of follow-up check-in survey
  - Updated news about university enrollment, visa policy, or activities
  - Artificial intelligence–supported tool (eg, chatbot)
  - User profile
  - More introductions on the dashboard page
- Current strengths
  - Easy to navigate the feedback and complete the check-in questions
  - Easy to switch language
  - Good page design (eg, color)

**Recommendation content improvement**

- Areas to improve
  - More instructions on how to read and use the recommendations
  - Recommendations could be more specific

- Current strengths
  - Feedback is comprehensive
  - Recommendations provided are useful

From the design workshops, participants indicated that the key value of the platform is addressing cross-cultural challenges by offering surveys and recommendations that cover many of the daily life issues faced by Chinese-speaking international students, along with language selection options. However, areas for improvement include some alternative or transformative ideas on the content and engagement aspects, such as covering a broader and more diverse range of daily challenges, providing more individualized and specific recommendations, and incorporating interactive features, such as an artificial intelligence chatbot and discussion forum.

#### Phase 2 Design Insights and Strategies

The Phase 2 workshops demonstrated how students are engaged with the prototype built from Phase 1 findings and highlighted further refinements needed for impact. Participants valued the tool’s focus on cross-cultural challenges, the inclusion of DASS-21 and daily life circumstances, and the accessible, multilingual interface. This confirmed that grounding support in everyday challenges was a meaningful entry point for Chinese-speaking international students.

Meanwhile, participants identified gaps. Survey content was seen as too narrow and occasionally difficult to understand, with calls to expand coverage to career planning, financial management, collaboration, family commitments, and visa issues. Recommendations were described as useful but overly general, with a need for clearer guidance on how to apply them.

Finally, students emphasized the importance of interactivity and personalization. Suggested features included an artificial intelligence chatbot, peer discussion forum, follow-up surveys, and practical updates on enrollment and visa policies. These insights extend the Phase 1 findings by highlighting that effective tools must move beyond awareness-building to provide specific, actionable, and engagement supports that fit students’ daily realities.

## Discussion

### Principal Findings

This study explored needs and challenges for developing a digital tool to support the mental health of Chinese-speaking international students based on interviews and workshops conducted over 2 phases. Overall, findings revealed significant gaps in the students’ mental health awareness, literacy, and help-seeking, with likely internalized stigma toward mental health issues and help-seeking that further prevents preventative and interventive engagement with mental health. It is also evident that studying and living abroad means that students are experiencing elevated risk factors, such as acute and often ongoing stress, uncertainty, and lack of support, while having limited access to reliable, culturally tailored resources for their preferred support mechanism—self-help. In response, the study proposes design implications aimed at addressing these challenges through integrated self-help techniques and culturally relevant, evidence-based resources aimed at (1) raising mental health awareness, (2) building mental health literacy, and (3) encouraging proactive help-seeking and access to health services if mental health risk factors emerge. The following sections will elaborate on these key implications separately.

### Bridging Lived Experience and Mental Health Awareness Through Challenges

This study shows that while students identified daily life challenges created by the acute geographical transition with ease, they have difficulties recognizing the mental health implications that are associated with these elevated challenges. For example, they have identified challenges associated with cross-cultural adaptation, demanding and often overwhelming course workloads, and navigating a new social and independent lifestyle, and they have articulated the negative emotions and mood changes (“emo”) associated with these challenges with clarity. However, they did not seem to interpret these experiences and feelings as possible warning signs of mental health illness [38]. Recognition of early risk factors and symptoms of mental health issues is considered fundamental in mental health awareness, which aids identification, management, and prevention of mental health issues [42,43]. A lack of recognition of early warning signs is consistent with previous literature [44,45] reporting that lacking awareness is a key barrier in university students seeking help, which could then lead to serious consequences, including the development of severe mental health disorders [46] and suicidal behaviors [47].

To address this challenge, a mental health support system for Chinese-speaking international students’ needs to “build a bridge” by contextualizing the development of mental health awareness that the students need to build in the everyday challenges that the students tend to recognize themselves. This could be supported by a self-directed check-in mechanism [48-51], where students are invited to fill out surveys and questionnaires that cover concrete and shared academic and daily life challenges, cross-cultural difficulties, and mental health measures (eg, DASS-21). Through this comprehensive self-assessment check-in tool, students are provided insights into the main areas of significant everyday life challenges while being engaged in the mental health implications of these experiences and challenges, which leverage promising effects on increasing awareness of mental health issues, particularly in young people, where multiple studies have observed improved self-efficacy [52-55]. To strategically demonstrate the results of the self-assessment tool, the system should integrate the categories of challenges with associated mental health implications when visualizing the results.

It is also essential to address language barriers with a culturally sensitive adaptation approach [56], where measurement tools validated in Chinese contexts and mindful symptom descriptions are used to engage students. Consistent with prior research [57-61], our findings show that language barriers are significant barriers that affect most domains of international students’ lives. Language selection options have been identified as crucial in digital mental health services and interventions to effectively serve diverse user populations [62]. A study on digital mental health platforms for Chinese-speaking international students found that offering both simplified and traditional Chinese options significantly improved student engagement with the programs [19]. Importantly, adapting an evidence-based digital tool to a new language often requires appropriate cultural appropriations instead of a direct translation [63]. Therefore, using validated Chinese versions of scales or intervention programs (eg, the Chinese Short Version of the Depression Anxiety and Stress Scale [64] to assess students’ mental health conditions is essential.

### Mental Health Literacy Building Through Culturally Adapted Resource

In addition to a less-than-ideal mental health awareness, this study also identified a common tendency of underdeveloped mental health literacy (MHL), which manifested in insufficient and often incoherent knowledge regarding the causes, recognition, duration, and severity of various mental health conditions among the students. The data also revealed limited access to educational resources and, at times, reliance on ineffective self-management strategies to cope with negative emotions. This limited MHL, which has been defined as “both knowledge and beliefs about mental disorders, assisting in their recognition, management, or prevention” [39], plays a significant role in mental health prevention while contributing to better decision-making and increasing help-seeking in the context of mental health [65-67]. Our findings also showed that students’ perception of the host country’s (ie, Australia) attitude is one that is more open and understanding in contrast to their country of origin. This perceived openness led them to believe that there are more resources and services available; however, little was articulated as to how they would make use of these resources. This is largely due to the language barriers, cultural concerns (eg, worries of not being understood), and negative perceptions about the relevant resources (eg, mental health services are not helpful).

To address the challenge of underdeveloped MHL complicated by culturally based stigma, Jorm et al [39] framework for increasing MHL can provide a conceptual base that strategically focuses on developing students’ recognition and beliefs about mental health. However, for a culturally appropriate intervention for Chinese-speaking international students, integration of culturally informed design consideration is essential to address the unique challenges demonstrated in the findings. Key design considerations can include (1) normalizing emotional challenges as shared experiences in the student community [68], (2) enabling anonymous peer stories, examples, and narratives to foster a sense of group belonging and culturally familiar support [69], and (3) maintaining a high level of confidentiality while encouraging self-help through self-directed content without risk of stigmatization [70].

To further operationalize these key design considerations, the culturally sensitive approach will entail incorporating culturally related ways of expression and educational or health resources in designing the mental health technologies [71,72]. The key is to identify culturally appropriate ways to proactively reflect and ameliorate stigma (eg, “Mental health issues are often seen as just complaining without real problems”; P9), cultural biases (eg, the unique way Chinese-speaking international students describe mood swings as “emo”), and limited understanding or knowledge about the local health care systems and social norms. Personalization mechanisms will also be used to enable students to gain knowledge that can help them to interpret their own results while relating to MHL. Importantly, rather than presenting a matter-of-fact type diagnosis, the system would provide feedback to students with a culturally informed sense of empathy, linking the results to the challenges that they experience to normalize the results that the student might find too challenging to accept. In addition, the feedback will be linked to recommendations or resources that align with the students’ interests and needs, particularly those related to cultural aspects, such as language support, peer support, and assistance with geographical transitions.

### Encouraging Professional Help-Seeking

This study found that Chinese-speaking international students shared a limited level of help-seeking intentions where they feel that professional help is “something for other people” or for “very severe cases.” This is consistent with prior work reporting that international students are resistant toward counselors and counseling services [73]. This resistance persists even when participants experience emotional challenges, such as feeling “emo,” leading to a preference for solitude and self-coping. Recent studies have indicated that help-seeking intentions are positively correlated with better well-being outcomes among university students [74], especially international students whose help-seeking intentions subsequently influence their levels of psychological distress [75]. However, important barriers were reported by prior work [76], suggesting that international students tend to be inexperienced with professional psychological help, which then leads to negative attitudes toward these services (eg, perceiving these services as untrustworthy and inappropriate for solving personal difficulties). In line with prior work, various barriers to seeking professional help were identified in this study, including language and cultural barriers, financial concerns, fear of judgment, and a lack of knowledge and experience with the health care system in Australia.

To design support that addresses the resistant attitude toward professional service and encourages students who experience emerging mental health issues to seek professional support, it is key to “demystify” the process of seeking and receiving professional support and build a culturally appropriate “roadmap” to professional help. This can be operationalized by (1) providing peer voice (eg, lived experience examples) and voice from culturally tailored mental health services, (2) encouraging students to take the first step in help seeking, and (3) supporting students to navigate the initial contact point and then into the broader health care system while providing language, culture, financial information, and emotional support to overcome the specific barriers as articulated in the findings.

The system should provide destigmatizing mechanisms through peer stories for students to feel safe and reassured to take the first step toward accessing the health care services [77]. Relevant lived experience stories of other Chinese-speaking international students can be integrated here to reduce perceived language and cultural barriers and social stigma while showcasing the effects and benefits of professional support. In addition, providing information that demonstrates, in a linguistically and culturally sensitive way, why help-seeking is necessary and beneficial and how services protect the privacy of help-seekers and ways that help can be sought “here and now” without monetary concerns. This would entail listing available free services tailored to the students’ preferences, interpretation services coupled with various free helplines, and what specific areas these helplines provide support on. This “in the moment” education could significantly increase the students’ familiarity with professional help and aid their decision-making processes, which increases the chances of them taking the first step.

For students who are at an elevated risk of mental health problems, seeking and obtaining mid- to long-term professional support is necessary. For this group of at-risk students, it is imperative to guide and empower them to contact emergency services and develop the ability to go beyond the initial step and access the broader allied health care system for support of an ongoing nature. The design here should focus on accompanying students to build their own care pathway by providing culturally tailored guidelines and resources on available services and immediate steps. This can include a “one-stop-shop” toolkit of specific functions with contact and action suggestions at each step, such as (1) navigating the Australian health care system, (2) identifying clinicians with appropriate backgrounds and experiences, (3) identifying and using available financial support, and (4) understanding what happens in a therapy session. As student and social support initiatives that introduce educational resources or social skills have shown benefits in strengthening self-efficacy and confidence [78,79], this toolkit design will not only rapidly increase students’ relevant knowledge but also build their confidence and ability to navigate and make use of support that is available to them.

It is important to note that the universities are often the first place students turn to for help. It is essential for educational institutions to play a central role in introducing or providing a digital mental health support service. This study identified multiple challenges faced by international students, many of which are closely related to their academic experiences. In addition, a significant limitation of university services was also observed in our study and prior work. A critical analysis of Australian university mental health policies for international students found that university policies often frame distress as an individual concern, placing the focus and responsibility on students to be resilient, rather than providing much-needed support [13]. Prior research also found that the awareness of on-campus mental health services can have a positive influence on international students’ help-seeking attitude and behavior [7] and that university-based information and digital tools have been proven effective in delivering these services to students [63,80]. While it is crucial for educational institutions to provide information on the availability of on-campus support, this is often not culturally and linguistically adapted. In this case, the designed tool should consider implementation possibilities for universities to adopt and use the digital tool.

### Limitations and Future Work

There are a few limitations of this study. Recruitment relied on internet-based recruitment that invited students “interested in addressing their mental health concerns,” which likely introduced self-selection bias toward participants who were more willing to discuss mental health. Perspectives of more avoidant or help-resistant students may therefore be underrepresented. The qualitative design and the relatively small, Chinese-speaking sample constrain statistical generalizability to the broader international-student population. Interpretation was situated within existing literature to support transferability. The disciplinary composition was imbalanced, with approximately 40% (8/20) of participants enrolled in public health, which may have oriented feedback toward public-health framings of mental health and intervention design. Within this qualitative dataset, no clear or consistent patterns were observed by years in Australia or by mental health literacy. Variation appeared more related to students’ study backgrounds, whether in mental health or other disciplines. Future research should recruit a larger and more balanced cohort, use stratified sampling across disciplines and lengths of stay, and include validated measures of mental-health literacy to enable multivariable and mixed methods analyses that inform more individualized, targeted in-platform feedback.

The given scope of the study focused on Australia as an academic context, and expanding the geographic location may allow for different findings to emerge. The study scope also limited the possibility of exploring diverse cultures that international students come from; however, it is essential for having an exploratory study to dive deeper into 1 student group and learn how cultural adaptation can be designed and developed. Future work can explore other cultures and their influences on the international students’ mental health to uncover similarities and differences in the students’ lived experiences and the unique mental health challenges and needs.

### Conclusions

In summary, a set of clear insights and solutions for designing mental health support for Chinese-speaking international students has been presented. This study adds insights to the digital tool development in relation to mental health support, with an understanding of the challenges and needs of this specific user group—international students. Current findings provide a rich and in-depth description of the barriers and needs associated with international students’ mental health while relocating to a new cultural context, and we have discussed how these needs and concerns are synthesized to inform the design implications. These design implications captured key mechanisms that enable culturally adapted strategies to help build international students’ mental health awareness and literacy while encouraging them to take actions toward professional help-seeking when needed. These design implications provide key considerations for researchers, designers, and developers who are interested in providing culturally sensitive and appropriate support for international students’ mental health and well-being.

## Supplementary material

10.2196/76695 Multimedia Appendix 1 Interview guide.

10.2196/76695 Multimedia Appendix 2 Workshop guide.

10.2196/76695 Multimedia Appendix 3 Interface examples.

## Abbreviations

DASS-21: Depression, Anxiety and Stress Scale–21 items
MHL: mental health literacy
REDCap: Research Electronic Data Capture
