# Supplementary material for: Designing Culturally Adapted Digital Mental Health Support Tool for Chinese-Speaking International Students in Australia: A Qualitative Co-design Study
Source: JMIR Form Res. 2025 Oct 21;9:e76695. doi: 10.2196/76695 (PMC12584277; doi:10.2196/76695)
Supplement: Multimedia Appendix 1 [file formative-v9-e76695-s001.docx]

**Appendix I**

**Interview Guide**

**Notes:**

- This is a guide only. Interviews will be semi-structured and tailored according to participant responses and situations.
- Interview questions will be translated into Chinese to accommodate participants’ preferences.
- Broadly, the interview will explore:
  - Views and attitudes toward mental health problems and help-seeking
  - Mental health-related experiences during study in Australia
  - Challenges in daily life (e.g., academics, accommodation, language, social connections, finances, lifestyle health)
  - Barriers and enablers to managing mental health problems
  - Barriers and enablers to accessing mental health services
  - Commonly used digital platforms for mental health information
  - Suggestions for features and content of a future online platform
- Interview duration: approximately **60 minutes**.

**1. Introduction to the purpose and content of the interview**
A. Thank the participant for joining and recap the consent form and explanatory statement signed prior to the interview.
B. Remind the participant they may withdraw at any time or skip any question they do not wish to answer.
C. Confirm their understanding of the explanatory statement and reconfirm consent.
D. Begin recording.

**2. Understanding and perceptions of mental health** *(guiding questions)*
A. How do you define or understand “mental health”?
B. In your view, are mental health problems perceived differently in China compared to Australia?
C. As an international student, what mental health issues do you find most concerning during your studies?

**3. Experiences and challenges** *(guiding questions)*
A. What challenges have you faced in daily life that may affect your mental health? (Prompt: academics, accommodation, language, social connections, finances, lifestyle health)
B. How have you responded to these challenges?
C. What has helped you cope, and what has made it harder?

**4. Attitudes and behaviours toward help-seeking** *(guiding questions)*
A. What is your attitude toward seeking help for mental health concerns?
B. What types of support do you believe are most suitable for international students?
C. Have you ever sought help from mental health services? What was your experience like?

**5. Digital tools and information sources** *(guiding questions)*
A. Which digital platforms or online tools do you commonly use to access information about mental health?
B. What are your views on social media or online communities for mental health support?
C. What features or content would you like to see in a digital mental health support tool designed for international students?

**6. End of interview debrief**
A. Summarise and conclude the session.
B. Thank the participant for sharing their experiences and opinions.
C. Ask how they felt about the session.
D. If any distress is observed or reported, validate and normalise their feelings.
E. Remind them of available helpline numbers and encourage them to seek support from existing networks if needed.
F. Inform them that the gift voucher will be sent via email within one week.
G. Stop recording.
