# Supplementary material for: Designing Culturally Adapted Digital Mental Health Support Tool for Chinese-Speaking International Students in Australia: A Qualitative Co-design Study
Source: JMIR Form Res. 2025 Oct 21;9:e76695. doi: 10.2196/76695 (PMC12584277; doi:10.2196/76695)
Supplement: Multimedia Appendix 2 [file formative-v9-e76695-s002.docx]

**Appendix II**

**Design Workshop Guide**

**Part 1: Icebreaker and Workshop Introduction (5 minutes)**
Welcome participants and thank them for attending. Confirm that they have had access to the GMCI-C prototype for approximately one week. Explain that the purpose of the workshop is to gather feedback on their experience using the platform, including suggestions for improvement, missing content, and any elements they found uncomfortable.

Outline the session structure:

1. Icebreaker and introductions.
2. Group discussion of user experience topics.

Remind participants that, as per their consent, the session will be recorded, but no identifiable information will be used in reporting.

**Icebreaker Activity:** Each participant shares one technique they use when feeling down (e.g., “I like to listen to gentle music and have dessert”).

**Part 2: User Experience Feedback**
Conduct a brief walkthrough of the prototype to refresh participants’ reflections. Facilitate discussion using the following guiding questions:

1. What were your initial impressions after using the platform? What actions did you take immediately afterward?
2. What are your thoughts on the survey questions? Did they address your mental health concerns, or were there gaps?
3. How did you find the recommendations and feedback provided? Were they useful? Was any content missing?
4. What additional features or functions would you like to see?
5. How would you describe the platform’s interface in terms of usability and navigation?

**Part 3: Debrief**

- Summarise the main points discussed.
- Thank participants for their time and contributions.
- Invite final comments or reflections.
- Check in on participant wellbeing and provide helpline contact information if needed.
- Remind them about the delivery of gift vouchers.

Miro board activity example - Chinese

**
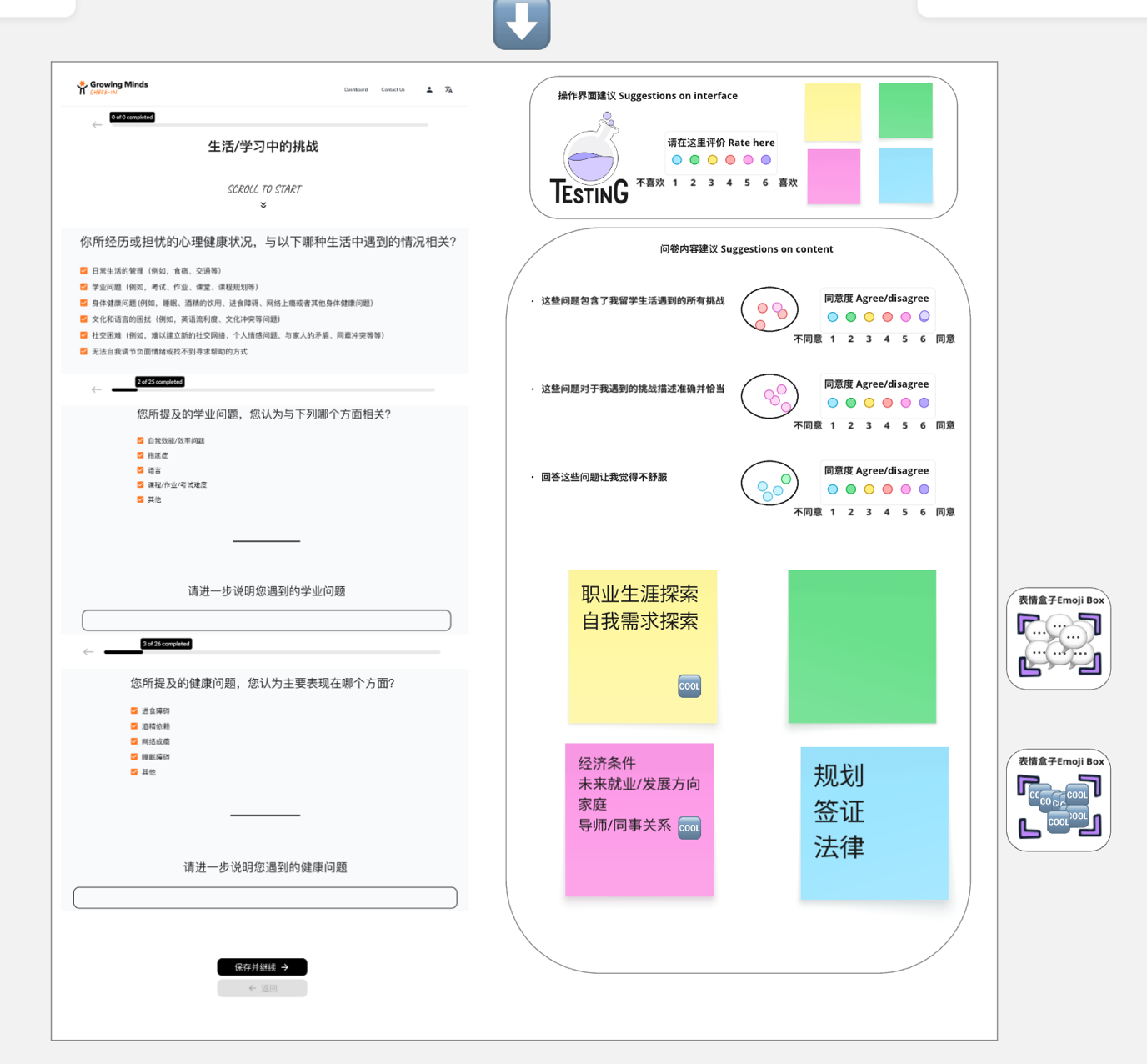
**
