# Supplementary figures and images for: Designing Culturally Adapted Digital Mental Health Support Tool for Chinese-Speaking International Students in Australia: A Qualitative Co-design Study
Source: JMIR Form Res. 2025 Oct 21;9:e76695. doi: 10.2196/76695 (PMC12584277; doi:10.2196/76695)

**Appendix III**

**
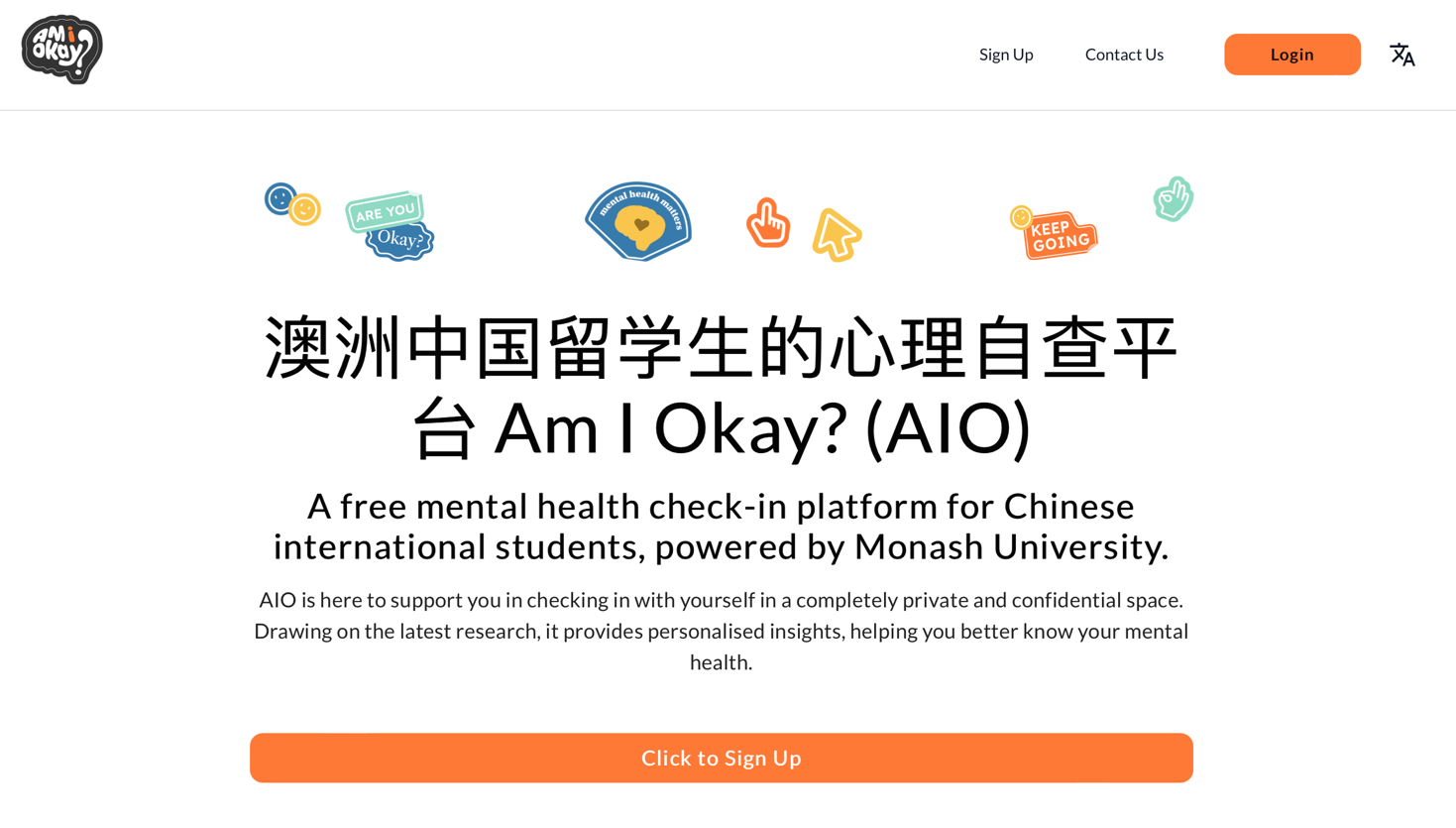
**

**
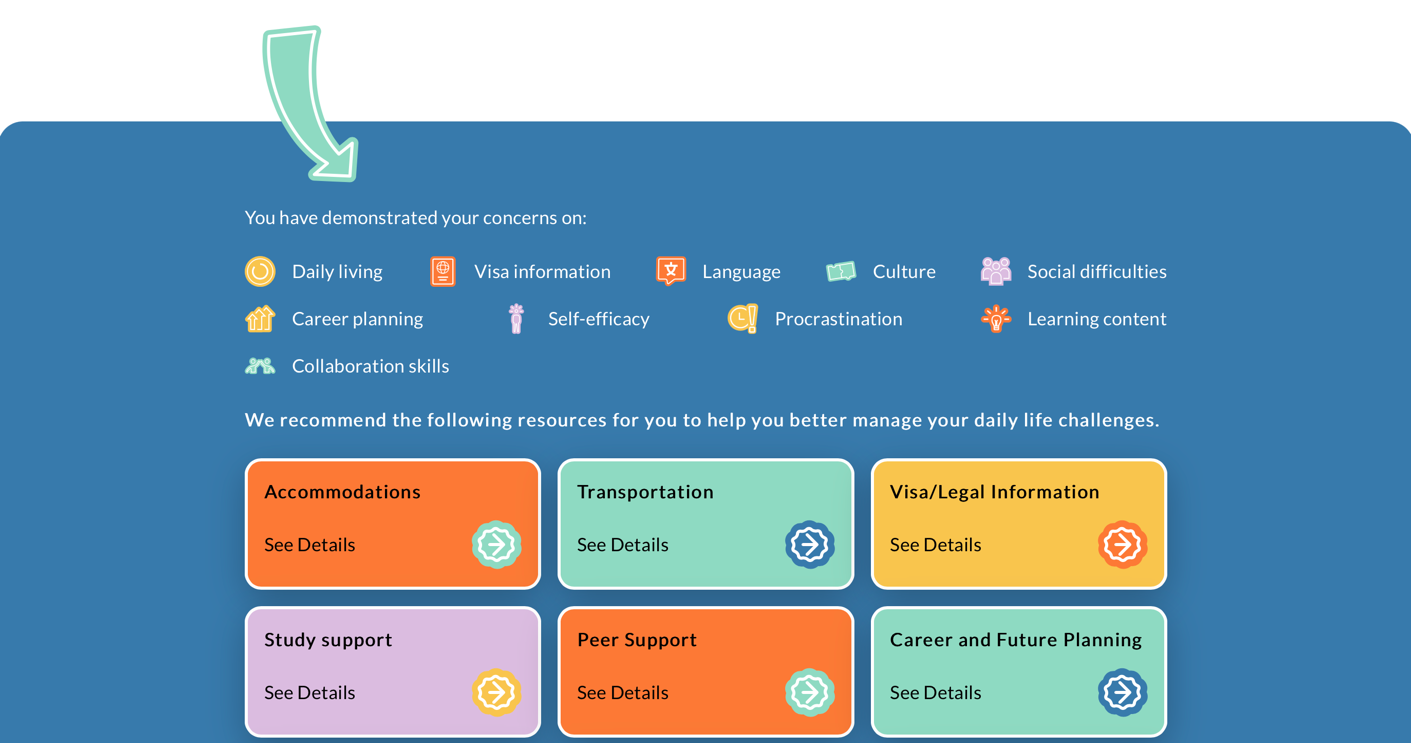
**

**
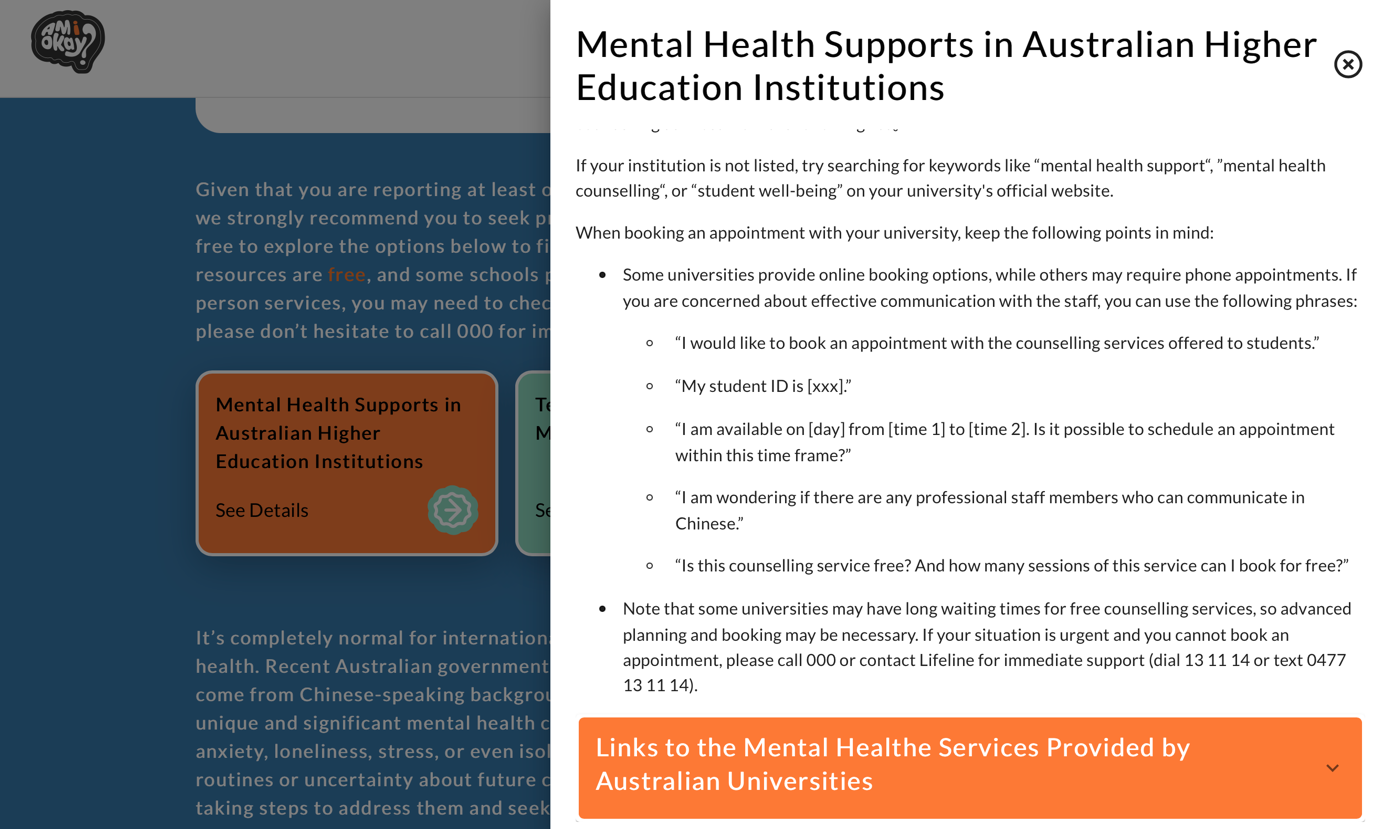
**

**
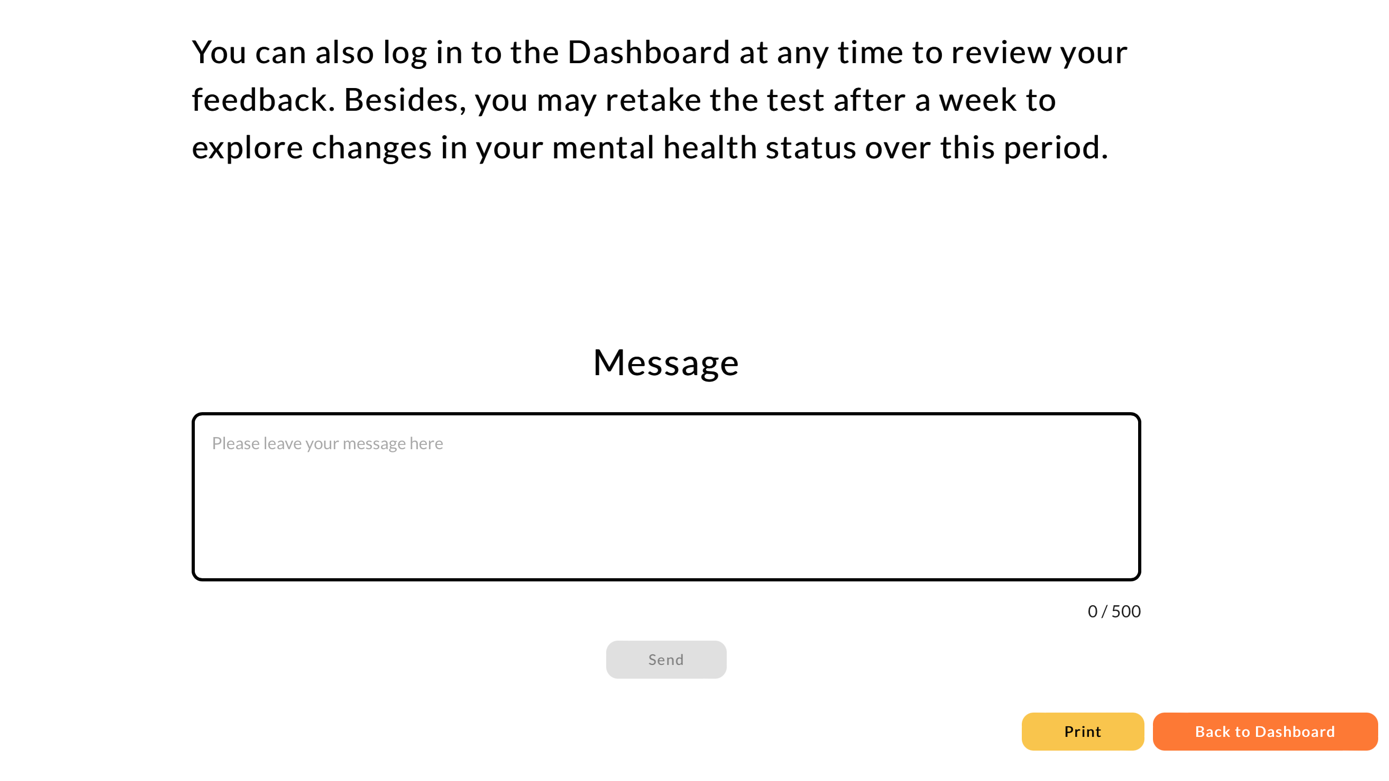
**

Supplement: Multimedia Appendix 3 [file formative-v9-e76695-s003.docx]
